# Variations in Cellular Responses of Mouse T Cells to Adenosine-5′-Triphosphate Stimulation Do Not Depend on P2X7 Receptor Expression Levels but on Their Activation and Differentiation Stage

**DOI:** 10.3389/fimmu.2018.00360

**Published:** 2018-02-27

**Authors:** Hanaa Safya, Amine Mellouk, Julie Legrand, Sylvain M. Le Gall, Mohcine Benbijja, Colette Kanellopoulos-Langevin, Jean M. Kanellopoulos, Pierre Bobé

**Affiliations:** ^1^UMR1174, INSERM, Université Paris-Sud, Orsay, France; ^2^Institut André Lwoff, CNRS, Université Paris-Sud, Villejuif, France; ^3^UMR 970, INSERM, Université Paris Descartes, Paris, France; ^4^UMR 1012, INSERM, Université Paris-Sud, Le Kremlin Bicêtre, France; ^5^UMR 1149, INSERM, Université Paris Diderot, Paris, France; ^6^UMR 9198, I2BC, CNRS, Université Paris-Sud, Orsay, France

**Keywords:** P2X7, CD39, CD73, regulatory T lymphocyte, CD62L shedding, pore formation, phosphatidyslerine exposure, cell death

## Abstract

A previous report has shown that regulatory T cells (Treg) were markedly more sensitive to adenosine-5′-triphosphate (ATP) than conventional T cells (Tconv). Another one has shown that Tregs and CD45RB^low^ Tconvs, but not CD45RB^high^ Tconvs, displayed similar high sensitivity to ATP. We have previously reported that CD45RB^low^ Tconvs expressing B220/CD45RABC molecules in a pre-apoptotic stage are resistant to ATP stimulation due to the loss of P2X7 receptor (P2X7R) membrane expression. To gain a clearer picture on T-cell sensitivity to ATP, we have quantified four different cellular activities triggered by ATP in mouse T cells at different stages of activation/differentiation, in correlation with levels of P2X7R membrane expression. P2X7R expression significantly increases on Tconvs during differentiation from naive CD45RB^high^CD44^low^ to effector/memory CD45RB^low^CD44^high^ stage. Maximum levels of upregulation are reached on recently activated CD69^+^ naive and memory Tconvs. Ectonucleotidases CD39 and CD73 expression levels increase in parallel with those of P2X7R. Recently activated CD69^+^ CD45RB^high^CD44^low^ Tconvs, although expressing high levels of P2X7R, fail to cleave homing receptor CD62L after ATP treatment, but efficiently form pores and externalize phosphatidylserine (PS). In contrast, naive CD45RB^high^CD44^low^ Tconvs cleave CD62L with high efficiency although they express a lower level of P2X7, thus suggesting that P2X7R levels are not a limiting factor for signaling ATP-induced cellular responses. Contrary to common assumption, P2X7R-mediated cellular activities in mouse Tconvs are not triggered in an all-or-none manner, but depend on their stage of activation/differentiation. Compared to CD45RB^low^ Tconvs, CD45RB^low^Foxp3^+^ Tregs show significantly higher levels of P2X7R membrane expression and of sensitivity to ATP as evidenced by their high levels of CD62L shedding, pore formation and PS externalization observed after ATP treatment. In summary, the different abilities of ATP-treated Tconvs to form pore or cleave CD62L depending on their activation and differentiation state suggests that P2X7R signaling varies according to the physiological role of T convs during antigen activation in secondary lymphoid organs or trafficking to inflammatory sites.

## Introduction

The P2X7 receptor (P2X7R) belongs to the P2X receptor family of adenosine-5′-triphosphate (ATP)-gated cation channels. The type of molecular and cellular responses induced by P2X7R depends on the length of stimulation by 0.5–1 mM concentrations of ATP in its tetra-anionic form (ATP^4−^) (1). Since P2X7R plays a major role in both innate and adaptive immunity, its involvement in the development of inflammatory and autoimmune diseases is extensively studied. Moreover, there is an increasing interest in the potential of P2X7R antagonists to treat a variety of inflammatory conditions (2). However, protective or detrimental effects of P2X7R on disease onset and/or development have been observed (3, 4). These seemingly contradictory reports emphasize the need for an in-depth investigation on how the various cellular functions triggered by the ATP/P2X7R signaling pathway are regulated in immune cells under normal and pathological situations. Brief stimulation of P2X7R induces cation-specific ion channels formation (5) and phosphatidylserine (PS) exposure in the plasma membrane (6). Prolonged activation of P2X7R results in the formation of nonselective membrane pores, permeable to molecules with a molecular mass up to 900 Da. Continuous activation of P2X7R can lead to cell death by apoptosis (7–9) and/or necrosis (10, 11), depending on the cell type. In contrast, P2X7R may trigger an anti-apoptotic or growth promoting activity (12, 13). Numerous physiological functions have been attributed to P2X7R; notably, activation of caspase-1 (14, 15), maturation and secretion of cytokines such as IL-1β, IL-18, IL-6, and TNF-α (3, 14, 16–18), migration of leukocytes (19), and killing of intracellular pathogens in macrophages (20, 21). Moreover, P2X7R activation triggers proteolytic cleavage of membrane proteins such as the homing receptor L-selectin (CD62L), the low affinity receptor for IgE (CD23) (22, 23), TNF-α (24), IL-6 receptor (25), and the amyloid precursor protein (26). P2X7R also regulates the early signaling events involved in T-cell activation. Upon antigen stimulation, T lymphocytes release ATP, which induces Ca^2+^ influx, NF-AT activation, and IL-2 production through P2X7R activation (27–30). Moreover, ATP plays a crucial role in regulating the differentiation of CD4^+^ T cells into Th17 cells (31, 32). During chronic inflammation, ATP could also facilitate the conversion of regulatory CD4^+^ T cells (Treg) into Th17 cells (33). Sensitivity to ATP varies among different T-cell subpopulations. Thus, CD8^+^ T cells from the spleen, lymph nodes, or liver exhibit low levels of P2X7R membrane expression and ATP sensitivity, whereas both are displayed at high levels in intestinal CD8^+^ T cells (34). Concerning CD4 Tregs, although significantly higher levels of *p*2X7 mRNA were found in CD4^+^ Tregs compared to CD4^+^ conventional T lymphocyte (Tconvs) (33), contradictory reports have been published about their sensitivity to P2X7R-induced cell death. In one report, CD25^+^CD4^+^ Tregs are markedly more sensitive to P2X7R stimulation than CD25^−^CD4^+^ Tconv (35). In another, the sensitivity of CD4^+^ Tregs to P2X7R stimulation is normal and similar to that of CD4^+^ Tconvs, provided that the latter express, like Tregs, low levels of the RB isoform of the transmembrane tyrosine phosphatase CD45 (CD45RB) (36). In Tconvs, the sensitivity to ATP-induced PS externalization and cell death appears to inversely correlate with the levels of CD45RB membrane expression. Thus, CD4^+^ T cells expressing low levels of CD45RB (CD45RB^low^) are significantly more sensitive to ATP stimulation than their counterpart expressing high levels of CD45RB (CD45RB^high^) (36, 37). However, the levels of P2X7R membrane expression were not determined in these studies. Moreover, we have shown that effector T lymphocytes become totally resistant to P2X7R stimulation following the plasma membrane expression of the B220 isoform of CD45 (or CD45RABC) (38) during the process of activation-induced cell death (39–41). The resistance of B220^+^ T lymphocytes to ATP stimulation is due to the loss of P2X7R expression at the plasma membrane, as it is retained in the cytosol (38).

High and low levels of CD45RB expression on mouse T cells are a feature of naive and antigen-activated cells, respectively (42). Therefore, one could conclude from previous reports (36, 37) that activated T cells (CD45RB^low^) are more sensitive to ATP-induced PS externalization and cell death than naive T cells (CD45RB^high^). However, we found that recently activated naive CD69^+^CD45RB^high^CD44^low^ Tconvs show a significantly reduced ability to proteolytically cleave CD62L compared to naive CD69^−^CD45RB^high^CD44^low^ T cells although they express higher levels of P2X7R. The reverse situation was observed for ATP-induced pore formation, and to a lesser extent for PS externalization, which were significantly upregulated in recently activated naive compared to naive Tconvs. To our knowledge, this is the first report describing a complete dissociation of ATP-induced cellular activities during the activation and/or differentiation of Tconvs, regardless of the levels of P2X7R and ectonucleotidases CD39 and CD73 membrane expression. Compared to CD45RB^low^ Tconvs, Foxp3^+^ Tregs that have an activated phenotype (CD45RB^low^ or CD25^+^) show higher levels of P2X7R membrane expression and of sensitivity to ATP. Thus, our present data show that the regulation of T cell sensitivity to ATP is far more complex than previously considered, as we found that P2X7R-mediated cellular activities in T-cell subsets are not dependent on the levels of P2X7R membrane expression and not triggered in an all-or-none manner. They depend on their stage of activation/differentiation.

## Materials and Methods

### Reagents

Adenosine-5′-triphosphate, phorbol myristate acetate (PMA), concanavalin A (ConA), EGTA, and KN-62 (1-[N,O-bis(5-Isoquinolinesulfonyl)-N-methyl-L-tyrosyl]-4-phenylpiperazine) were purchased from Sigma-Aldrich (St. Louis, MO, USA). YO-PRO-1 and YO-PRO-3 dyes and BAPTA-AM were from Life Technologies (Carlsbad, CA, USA). Metalloprotease inhibitor GM6001 was from Chemicon International (Temecula, CA, USA). ATP solutions were prepared extemporaneously from 100 mM stock solution (pH 7.4) stored at −20°C. Because divalent ions affect the potency of ATP^4−^ to bind P2X7R, the cell medium used to activate PX7R contains low concentrations of Mg^2+^.

### Mice

Wild-type C57BL/6J (B6) and *P2rx7* knockout B6.129P2-*P2rx7^tm1Gab^*/J (P2X7R KO) (16) mice originally from The Jackson Laboratory (Bar Harbor, ME, USA) were maintained in our animal facilities (CNRS SEAT UPS44, Villejuif, France and animalerie NeuroPSI, Orsay, France). B6.Cg-*Foxp3^tm1Mal^*/J (Foxp3GFP) (43) mice were kindly provided by Dr Géraldine Schlecht-Louf (INSERM UMR 996, France). All the experiments were conducted in accordance with French (décret n° 2013-118) and EU (directive 86/609/EEC) guidelines for the care of laboratory animals and approved by our local research ethics committee (CEEA 59).

### Flow Cytometry Immunophenotyping Assays

Spleen cell suspensions were phenotyped by flow cytometry using fluorescent-conjugated monoclonal antibody (mAb): anti-CD90.2/Thy1.2 (clone 30-H12), anti-B220 (clone RA3-6B2), anti-CD45RA (Clone 14.8), anti-CD45RB (clone C363.16A), anti-CD45RC (C363-16A), anti-CD4 (clone GK1.5), anti-CD69 (clone H1.2F3), anti-CD44 (clone IM7), anti-CD62L (clone MEL-14), anti-CD197/CC-chemokine receptor 7 (CCR7) (clone 4B12), CD39 (clone 24DMS1), and CD73 (clone TY/11.8) (all from eBioscience). P2X7R was detected using a rabbit polyclonal anti-P2X7R serum described in Le Gall et al. (38) and fluorescent-conjugated goat anti-rabbit IgG F(ab)′_2_ secondary antibodies (eBioscience). Fluorescent-conjugated rat IgG2a, IgG2b or Armenian hamster IgG mAbs were used as the isotype control (eBioscience). Use of mAb to mouse Fcγ receptor (eBioscience) avoided non-specific antibody binding. Data acquisition was performed at the Flow cytometry core facility at I2BC, CNRS UMR 9198.

### CD62L Shedding, PS Exposure, Pore Formation, and Cell Death Assays

Spleen cells suspended in RPMI 1640 medium (Invitrogen, France) were treated with ATP or PMA in a humidified 5% CO_2_ atmosphere at 37°C for 30 min or 2 h, depending on the assay. After washing with RPMI 1640 medium, cells were resuspended in FACS buffer (eBioscience) and stained for 30 min on ice with phenotype-specific fluorescent mAbs and fluorescent-conjugated anti-CD62L mAb to assess CD62L shedding. PS cell surface exposure was detected on mAb-labeled cells using FITC- or PE-Annexin V apoptosis detection kit according to the manufacturer’s specifications (eBioscience, France). To quantify P2X7R-mediated pore formation, ATP treatment was performed in the presence of either the green-fluorescent YO-PRO-1 (molecular weight 629 Da) or the orange-fluorescent YO-PRO-3 (molecular weight 655 Da) nucleic acid dyes, depending on the fluorochromes used in the phenotyping step. Cell morphology (FSC/SSC) and Annexin V staining were used to quantify dead/dying cells (Annexin V^+^ FSC^low^ SSC^high^) by flow cytometry. In some experiments, cells were pretreated with metalloprotease inhibitor GM6001, P2X7R antagonist KN-62, intracellular calcium chelator BAPTA-AM (10 µM) or extracellular calcium chelator EGTA (5 mM) for 30 min at 37°C with 5% CO_2_ prior treatment with ATP or PMA.

### Transfection and Flow Cytometry Assays

The COS7 epithelial cell line was transfected transiently with a pCDEF3 expression vector containing CD45RABC cDNA (kindly provided by Dr A. Weiss, UCSF, San Francisco, CA, USA). At 48 h after transfection, the cells were stained with FITC-conjugated anti-CD45RA (clone 14.8), PE-conjugated anti-CD45RB (clone 16A), APC-conjugated anti-CD45RC (clone GL24), and PE Cy5.5-conjugated anti-CD45RABC (clone RA3-6B2) mAbs, and analyzed by flow cytometry.

### Statistical Analysis

Data are reported as mean ± SEM. Comparisons between untreated and treated groups were made by Student’s *t*-test. Degrees of significance are indicated as follows: *p ≤ 0.05, **p ≤ 0.01, ***p ≤ 0.001.

## Results

### ATP-Mediated Cellular Activities and P2X7R Membrane Expression in T Cells with either High or Low Expression of CD45RB

**Figure 1:**
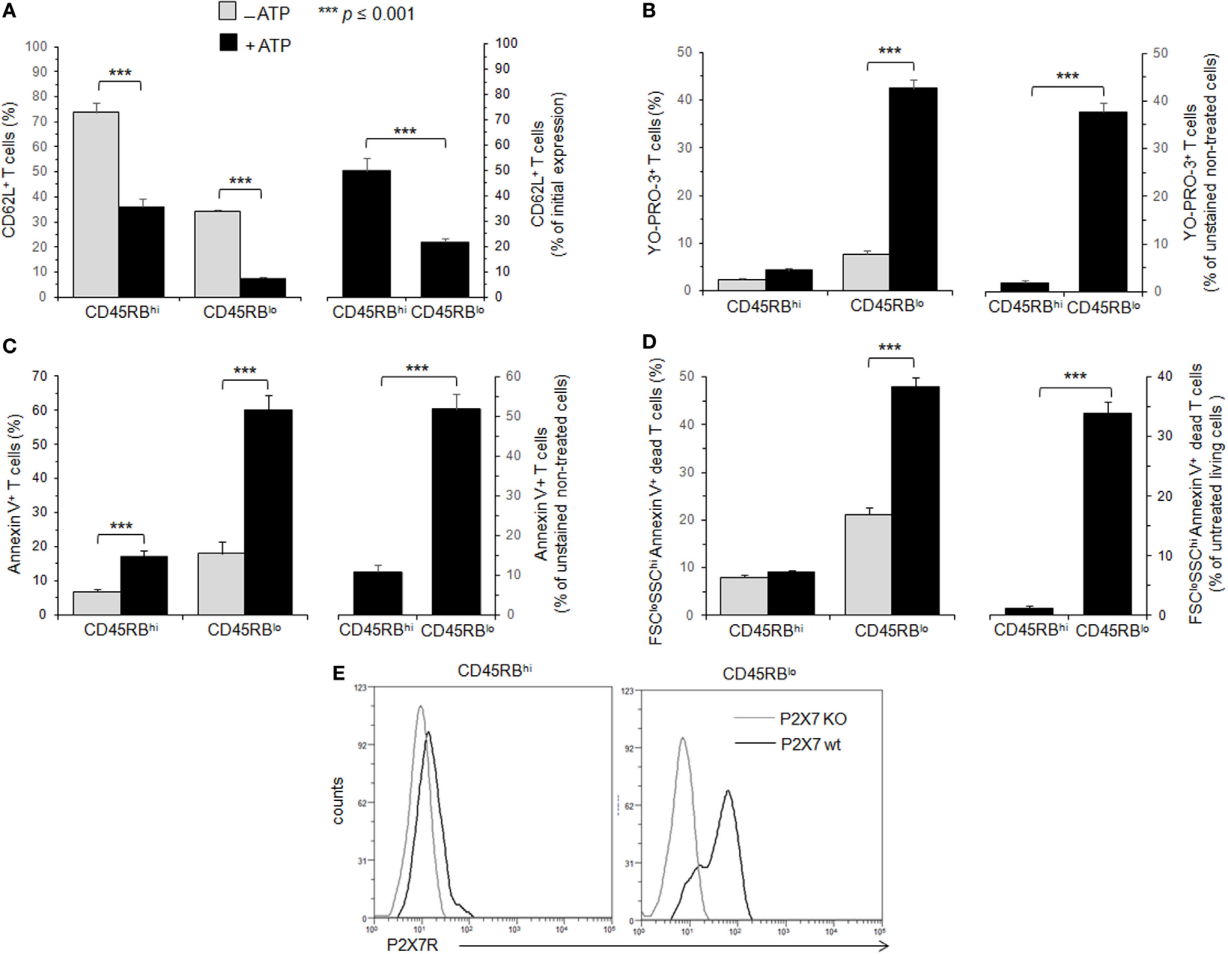
Adenosine-5′-triphosphate (ATP)-mediated cellular activities and P2X7 receptor (P2X7R) membrane expression in T cells with either high or low CD45RB phenotype. **(A–D)** Spleen cells from B6 mice were either left unstimulated or stimulated with 500 µM ATP for 30 min **(A–C)** or 2 h **(D)** in the presence or absence of YO-PRO-3 fluorescent probe. Cells were subsequently stained with fluorescent monoclonal antibodies (mAbs) against phenotypic markers CD90, B220, CD4, CD45RB, and CD62L as well as Annexin V fluorescent probe. CD62L shedding, pore formation, phosphatidylserine (PS) exposure, or cell death were assessed within the gated CD45RB^high (hi)^ or CD45RB^low (lo)^ CD90^+^CD4^+^B220^−^ T-cell subsets by flow cytometry. Cell morphology (FSC/SSC) and Annexin V staining were used to quantify dead/dying cells (Annexin V^+^ FSC^lo^SSC^hi^). Results are expressed as the mean percentage ± SEM of CD62L^+^ cells **(A)**, YO-PRO-3^+^ cells **(B)**, Annexin V + FSC^hi^ SSC^lo^ living cells **(C)** or FSC^lo^SSC^hi^ Annexin V^+^ dead cells (**D**) in the presence (■) or absence (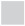) of ATP (left panels). For each cellular activity, the results are also expressed [(**A–D)**, right panels] as the ratio ± SEM between the percentages of cells expressing or non expressing cellular activities **(A–D)** in the presence or absence of ATP, respectively. Data are representative of at least six independent experiments with six to nine mice per group per experiment. Asterisks denote statistically significant differences between the indicated groups (***p ≤ 0.001). **(E)** P2X7R membrane expression on CD45RB^hi^ or CD45RB^lo^ CD90^+^CD4^+^B220^−^ T cells from wild-type and P2X7R KO mice was measured using rabbit polyclonal anti-P2X7R antiserum (1:100) and fluorescent-conjugated goat anti-rabbit IgG F(ab)′_2_ secondary antibodies. P2X7R-staining histograms of wild-type T-cell subsets (black histograms) are overlaid on P2X7R-staining histograms of P2X7R KO T-cell subsets (gray histograms). The histograms are representative of six individual mice.

Effector T cells express low levels of the CD45RB (42). Previously, we have shown that effector CD45RB^low^ T cells become resistant to ATP stimulation when they reach a preapoptotic stage characterized by the plasma membrane expression of B220 (or CD45RABC) (38). Therefore, reports (36, 37) showing that CD45RB^low^ effector T cells are notably more sensitive to BzATP-mediated PS exposure and cell death than CD45RB^high^ naive T cells appear contradictory to our previous findings (38). The different ligands (ATP vs. BzATP) used to activate P2X7R could explain the discrepancy between our data and those of Taylor et al. However, we favor the hypothesis that the anti-CD45RB mAb used to gate CD45RB^high^ T cells in these reports (36, 37) also detect B220^+^ (or CD45RABC^+^) T cells because the anti-CD45RB mAb (clone 16A) recognizes an exon B-dependent epitope (44). To test our hypothesis, CD45-negative COS7 epithelial cells were transfected with a plasmid vector encoding mouse CD45RABC (or B220) and stained with fluorescent anti-B220, anti-CD45RA, anti-CD45RB and anti-CD45RC mAbs. We observed that anti-CD45RB mAb recognized CD45RABC^+^ COS7 cells, but not untransfected COS7 cells (CD45RABC^−^), confirming that anti-CD45RB mAb cannot distinguish between CD45RB and CD45RABC isoforms. Likewise, anti-CD45RA and anti-CD45RC mAbs recognized CD45RABC^+^ COS7 cells (Figure S1 in Supplementary Material). Therefore, we have quantified ATP-mediated CD62L shedding, pore formation, PS exposure and cell death in B220-negative T cells with either high or low CD45RB cell surface expression (Figure 1). In agreement with previous studies (37), we found CD45RB^low^ T cells displayed higher sensitivity to ATP-mediated PS externalization than CD45RB^high^ (Figure 1C). Moreover, CD45RB^high^ T cells displayed markedly lower sensitivity to ATP-mediated CD62L shedding (Figure 1A), pore formation (Figure 1B) and cell death (Figure 1D) than CD45RB^low^ T cells. Altogether, our data suggest that P2X7R-mediated cellular activities are poorly triggered in naive CD45RB^high^ T cells compared to CD45RB^low^ activated T cells.

Membrane P2X7R levels on T cells could be a limiting factor for triggering the various cellular responses induced by ATP, and reduced levels of P2X7R membrane expression could account for the lower sensitivity of CD45RB^high^ naive T cells to ATP. Therefore, we quantified P2X7R on CD45RB^high^ and CD45RB^low^ T cells using a rabbit polyclonal anti-P2X7R serum and flow cytometry. We found that P2X7R is notably less expressed on naive CD45RB^high^ T cells than on activated CD45RB^low^ T cells (Figure 1E), suggesting that (1) ATP-mediated CD62L shedding, pore formation, PS externalization and cell death are triggered in the presence of high levels of P2X7R membrane expression, which are weakly express on the surface of naive T cells; (2) T-cell activation upregulates the expression of P2X7R making T cells able to perform all ATP-induced cellular activities.

### ATP-Mediated Cellular Activities and P2X7R Membrane Expression in Conventional and Tregs with CD45RB^low^ or CD25^+^ Phenotype

**Figure 2:**
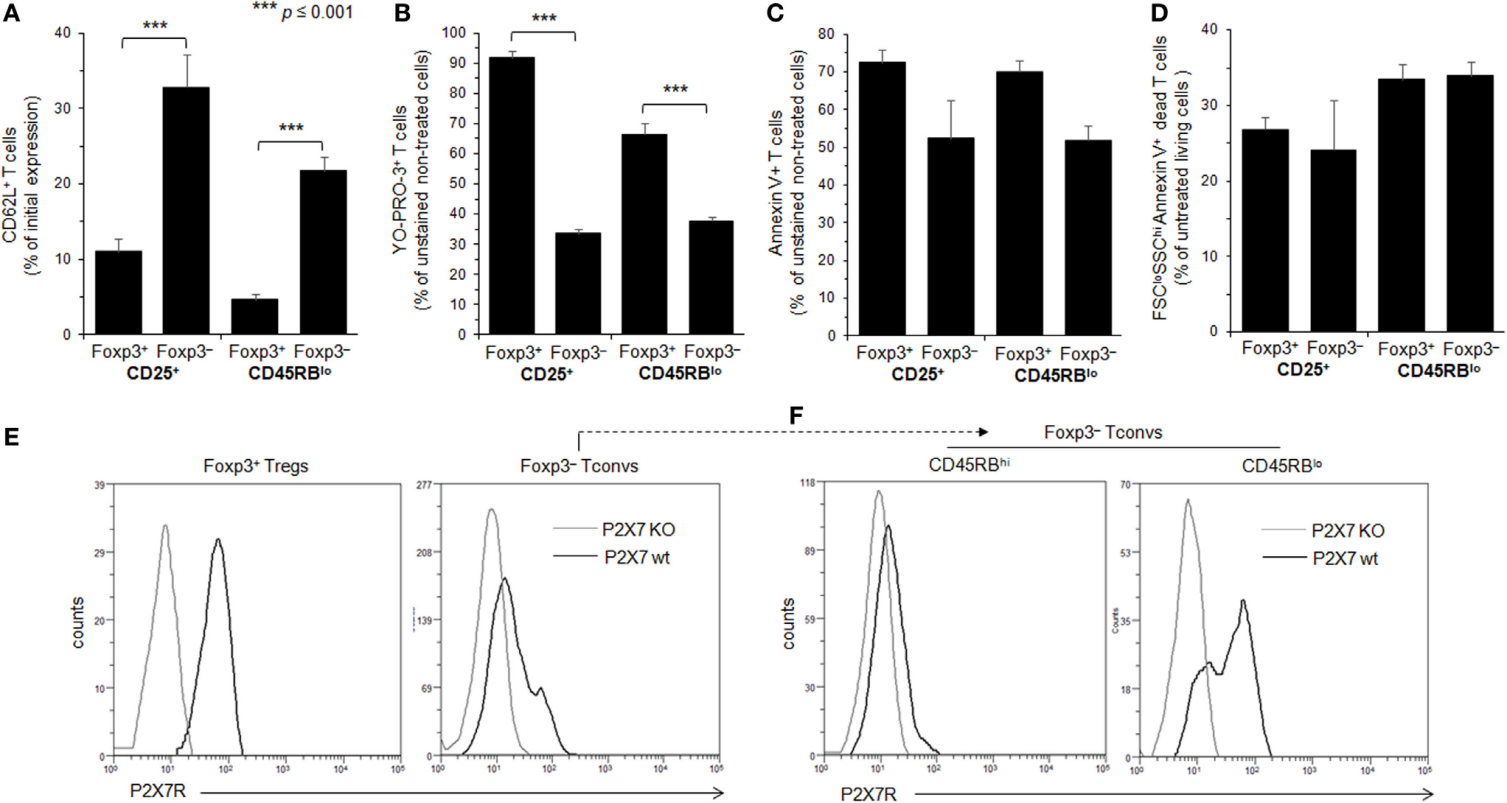
Adenosine-5′-triphosphate (ATP)-mediated cellular activities and P2X7 receptor (P2X7R) membrane expression in regulatory T lymphocytes (Tregs) and conventional T lymphocytes (Tconvs) with CD25^+^ and/or CD45RB^lo^ phenotype. **(A–D)** Spleen cells from Foxp3GFP B6 mice were either left unstimulated or stimulated with 500 µM ATP for 30 min **(A–C)** or 2 h **(D)** in the presence or absence of YO-PRO-3 fluorescent probe. Cells were subsequently stained with fluorescent monoclonal antibodies against phenotypic markers CD90, B220, CD4, CD45RB, and CD62L as well as Annexin V fluorescent probe. CD62L shedding, pore formation, phosphatidylserine (PS) exposure, or cell death were assessed by flow cytometry on gated GFP^+^ (Foxp3^+^) or GFP^−^ (Foxp3^−^) CD90^+^CD4^+^B220^−^ T cells with CD45RB^hi^, CD45RB^lo^, CD25^+^, or CD25^−^ phenotype. Cell morphology (FSC/SSC) and Annexin V staining were used to quantify dead/dying cells (Annexin V^+^ FSC^lo^SSC^hi^). Results on CD62L shedding **(A)**, pore formation **(B)**, PS exposure **(C)**, or cell death **(D)** are expressed as the ratio ± SEM (six mice per group) between the percentage of cells expressing or non expressing cellular activities **(A–D)** in the presence or absence of ATP, respectively. Data are representative of six independent experiments. Asterisks denote statistically significant differences between the indicated groups (***p ≤ 0.001). **(E,F)** P2X7R membrane expression on CD25^+^ or CD45RB^low^ Foxp3^+^ Tregs, and CD45RB^hi^ or CD45RB^lo^ CD90^+^CD4^+^B220^−^ Tconvs was measured using rabbit polyclonal anti-P2X7R antiserum (1:100) and fluorescent-conjugated goat anti-rabbit IgG F(ab)′_2_ secondary antibodies. P2X7R-staining histograms of wild-type T cells (black histograms) are overlaid on P2X7R-staining histograms of P2X7R KO T cells (gray histograms). The histograms are representative of at least 6 individual mice.

**Figure 3:**
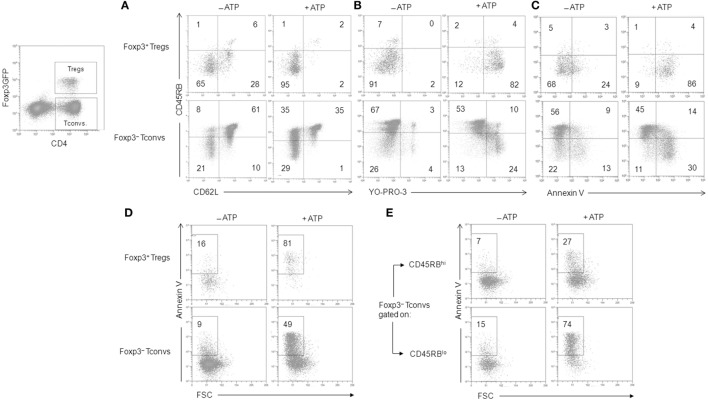
Adenosine-5′-triphosphate (ATP)-mediated cellular activities in regulatory T lymphocytes (Tregs) and conventional T lymphocytes (Tconvs). Spleen cells from Foxp3GFP B6 mice were either left unstimulated or stimulated with 500 µM ATP for 30 min **(A–C)** or 2 h **(D,E)** in the presence or absence of YO-PRO-3 fluorescent probe. Cells were subsequently stained with fluorescent monoclonal antibodies against phenotypic markers CD90, CD4, B220, CD45RB, and CD62L as well as Annexin V fluorescent probe. CD62L shedding **(A)**, pore formation **(B)**, PS exposure **(C)**, or cell death **(D,E)** were assessed by flow cytometry on gated GFP^+^ (Foxp3^+^) or GFP^−^ (Foxp3^−^) CD90^+^CD4^+^B220^−^ T cells with CD45RB^hi^ or CD45RB^lo^ phenotype. Numbers reported in the dot plots indicate the percentages of CD62L^+^ cells **(A)**, YO-PRO-3^+^ cells **(B)**, Annexin V^+^ FSC^hi^SSC^lo^ living cells **(C)**, or Annexin V^+^ FSC^lo^SSC^hi^ dead/dying cells **(D,E)** (either CD45RB^hi^ or CD45RB^lo^) in the gated CD90^+^CD4^+^B220^−^ T-cell population. Data are representative of six independent experiments with six mice per group per experiment.

The CD45RB^low^CD4^+^ T-cell subset encompasses conventional and Tregs (45). Different behaviors of Tconvs and Tregs upon ATP stimulation have been reported previously (35, 36). While one report concluded that Foxp3^+^CD25^+^CD4^+^ Tregs were markedly more sensitive to ATP-induced PS exposure and cell death than CD25^−^CD4^+^ Tconvs (35), another reported a similar susceptibility to ATP-induced PS exposure and cell death between CD4^+^ Tconvs and Tregs provided, however, that both T-cell subsets displayed the CD45RB^low^ phenotype (36). In an attempt to clarify these contradictory results, spleen cells from B6.Cg-*Foxp3^tm1Mal^*/J (Foxp3GFP) and P2X7R KO mice have been stimulated with ATP for 30 min or 2 h, and the levels of CD62L shedding, PS exposure, pore formation and cell death have been measured in CD4^+^ Tconvs (either CD25^+^ or CD45RB^low^) and Foxp3^+^CD4^+^ Tregs, which are mostly CD45RB^low^ (Figures 2 and 3). We found that CD25^+^Foxp3^+^ Tregs were three to four times more sensitive to ATP-mediated CD62L shedding (Figure 2A) and pore formation (Figure 2B) than activated CD25^+^Foxp3^−^ Tconvs. However, CD25^+^Foxp3^−^ Tconvs and CD25^+^Foxp3^+^ Tregs displayed similar sensitivity to ATP-mediated PS externalization (Figure 2C) and cell death (Figure 2D). When ATP sensitivity was analyzed in Foxp3^+^ Tregs and Foxp3^−^ Tconvs expressing a CD45RB^low^ phenotype, we found again a greater sensitivity of CD45RB^low^Foxp3^+^ Tregs to ATP-mediated CD62L shedding (Figures 2A and 3A) and pore formation (Figures 2B and 3B) compared to ATP-treated CD45RB^low^Foxp3^−^ Tconvs, but not to ATP-mediated PS externalization (Figures 2C and 3C) and cell death (Figures 2D and 3D,E) that was similarly high in CD45RB^low^ Tconvs and CD45RB^low^ Tregs. As expected, CD4^+^ T cells from P2X7R KO mice were totally resistant to ATP-induced cellular activities (data not shown). Altogether our results demonstrate a higher sensitivity of Tregs to P2X7R-mediated cellular activities compared to Tconvs.

The levels of P2X7R membrane expression are significantly higher on Foxp3^+^ Tregs than on Foxp3^−^ Tconvs (Figure 2E), and especially on CD45RB^high^ naive Tconvs (Figure 2F). Interestingly, and in contrast with Foxp3^+^ Tregs, P2X7R expression on CD45RB^low^ Tconvs is heterogeneous (Figure 2F). This would be in keeping with the phenotypic and functional heterogeneity of Tconvs and subsequently lead us to analyze the levels of P2X7R membrane expression and functions during their differentiation from naive to memory state.

### ATP-Mediated Cellular Activities and P2X7R Membrane Expression in Recently Activated Naive and Memory CD69^+^ T Cells

**Figure 4:**
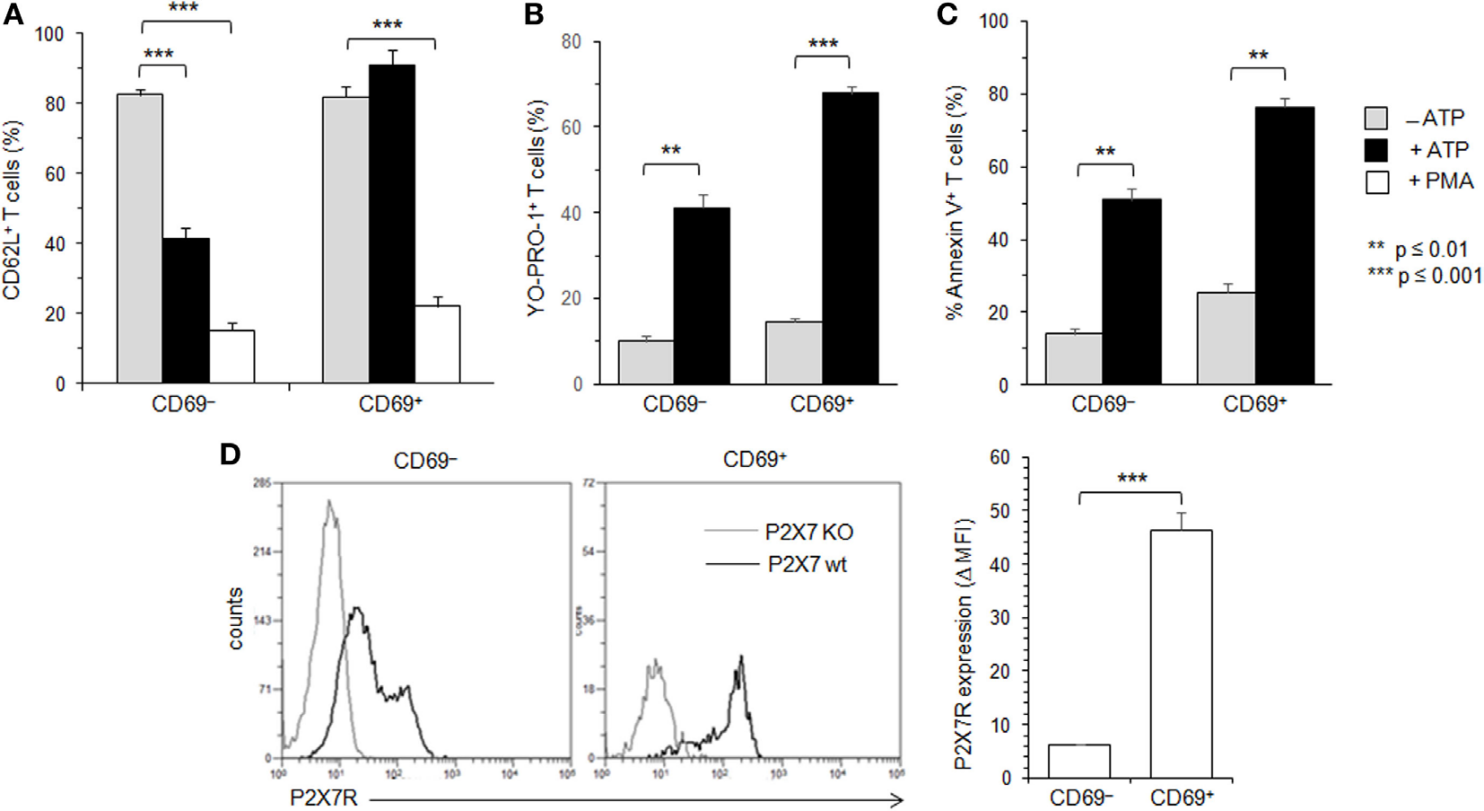
Adenosine-5′-triphosphate (ATP)-mediated cellular activities and P2X7 receptor (P2X7R) membrane expression in recently activated CD69^+^ conventional T lymphocytes (Tconvs). B6 mice were injected i.v. with 7 µg/g of T-cell mitogen concanavalin A for 18 h. Then, spleen cells were either left unstimulated or stimulated with 500 µM ATP or 25 ng/ml phorbol myristate acetate (PMA) for 30 min at 37°C with 5% CO_2_, in the presence or absence of YO-PRO-1 fluorescent probe. Cells were subsequently stained with fluorescent monoclonal antibodies (mAbs) against phenotypic markers CD90, CD4, B220, and CD69 as well as fluorescent-conjugated anti-CD62L mAb and Annexin V. CD62L shedding, pore formation, and phosphatidylserine (PS) exposure were assessed by flow cytometry on gated CD69^+^ or CD69^−^ CD90^+^CD4^+^B220^−^ Tconvs. Results on CD62L shedding **(A)**, pore formation **(B)**, or PS exposure **(C)** are expressed as the mean percentage ± SEM (*n* = 6–9 mice per group) of CD62L^+^, YO-PRO-1^+^, or Annexin V^+^ cells, after ATP or PMA stimulation. Data are representative of at least five independent experiments. **(D)** P2X7R membrane expression on CD69^+^ and CD69^−^ CD90^+^B220^−^ Tconvs was measured using rabbit polyclonal anti-P2X7R antiserum (1:100) and fluorescent-conjugated goat antirabbit IgG F(ab)′_2_ secondary antibodies. P2X7R-staining histograms of wild-type T-cell subset (black histograms) are overlaid on P2X7R-staining histograms of P2X7R KO T-cell subsets (gray histograms). Bar graph shows mean fluorescence intensity (MFI) ± SEM of P2X7R of 6 individual mice. Results are expressed as delta MFI (ΔMFI = MFI_wild type_ − MFI _KO_), i.e., change in MFI relative to P2X7R KO Tconvs. Asterisks denote statistically significant differences between the indicated groups (**p ≤ 0.01, ***p ≤ 0.001).

**Figure 5:**
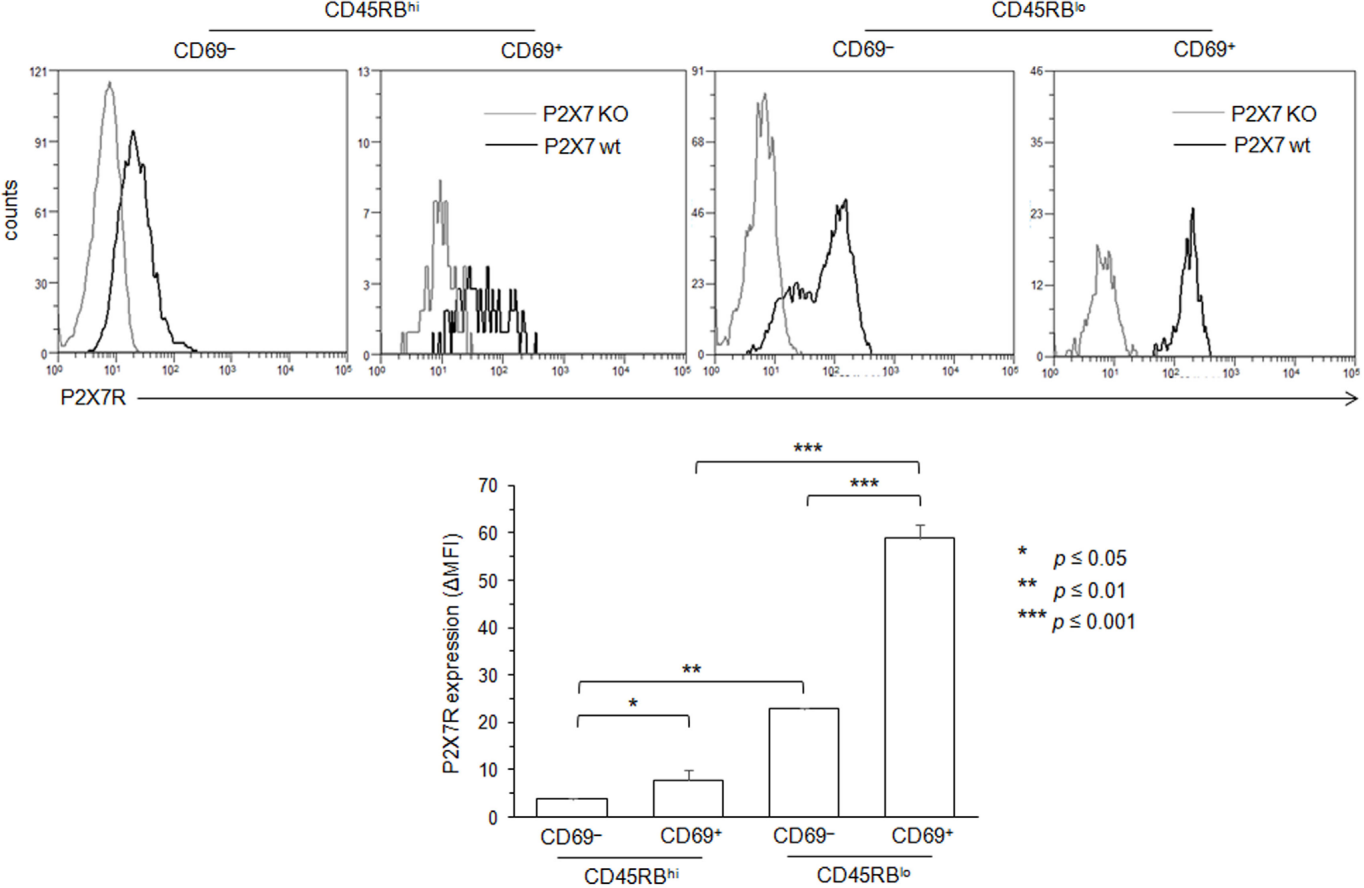
Variation in P2X7 receptor (P2X7R) membrane expression levels on conventional T lymphocytes (Tconvs) during differentiation from naive to effector/memory stage. P2X7R membrane expression on CD69^−^CD45RB^hi^ naive, CD69^+^CD45RB^hi^ recently activated naive, CD45RB^lo^ effector/memory or CD69^+^CD45RB^lo^ recently activated memory CD90^+^CD4^+^B220^−^ Tconvs from wild-type and P2X7R KO mice were measured using rabbit polyclonal anti-P2X7R antiserum (1:100) and fluorescent-conjugated goat anti-rabbit IgG F(ab)′_2_ secondary antibodies. In contrast with Figure 4, mice had not been injected with concanavalin A to maintain a high frequency of naive and memory resting T cells. P2X7R-staining histograms of wild-type T-cell subsets (black histograms) are overlaid on P2X7R-staining histograms of P2X7R KO T-cell subsets (gray histograms). The histograms are representative of at least 6 individual mice. Asterisks denote statistically significant differences between the indicated groups (*p ≤ 0.05; **p ≤ 0.01; ***p ≤ 0.001).

Figures 1–3 suggest that the cellular activities triggered by ATP correlate positively with the levels of P2X7R membrane expression. Moreover, these expression levels vary among Tconvs according to their state of activation and/or differentiation. Therefore, we have analyzed ATP-induced CD62L shedding, pore formation and PS exposure in B220^−^CD90^+^ Tconvs expressing CD69 (Figure 4), which is the earliest surface marker expressed during T-cell activation. The expression of CD69 marks the activation of naive and memory T cells. CD69 is expressed within < 4 h of activation and peak expression is between 18 and 48 h after stimulation (46). The percentage of T lymphocytes that express CD69 is usually low in the spleen of B6 mice (10.2 ± 2.8%, *n* = 10 mice). Therefore, B6 mice were injected intravenously with 7 µg/g of T-cell mitogen ConA for 18 h to massively increase the numbers of splenic CD69^+^ T cells (61 ± 7.5%, *n* = 14 mice) and their mean fluorescence intensity (MFI) (53 vs. 378). In contrast, the ConA treatment had no effect in both spleen cell number and the percentage of splenic T cells (Figure S2 in Supplementary Material). Unexpectedly, we found that CD69^+^ T cells, but not CD69^−^ T cells, failed to cleave their CD62L molecules after ATP treatment, both in terms of the percentages of CD62L^+^ T cells and levels of CD62L membrane expression (Figure 4A, and data not shown). In contrast, CD69^+^ T cells efficiently form pore (Figure 4B) and externalize PS (Figure 4C) after ATP treatment. The inability of recently activated CD69^+^ T cells to shed CD62L molecules was not related to defective proteolytic activity of ADAM-17 since both CD69^−^ and CD69^+^ T cells can shed CD62L after PMA treatment (Figure 4A). The expression of CD69 coincides with a strong upregulation of P2X7R membrane expression (Figure 4D) that is mainly observed on effector/memory CD45RB^low^ T cells (Figure 5).

In summary, recently activated CD69^+^CD45RB^low^ T cells, although expressing high levels of P2X7R, appeared mostly resistant to ATP-induced CD62L cleavage, indicating that P2X7R-mediated cellular activities are not triggered in an all-or-none manner in Tconvs and vary according to their stage of activation.

### ATP-Mediated Cellular Activities and P2X7R Membrane Expression in Effector/Memory CD45RB^low^CD44^high^ T Cells

**Figure 6:**
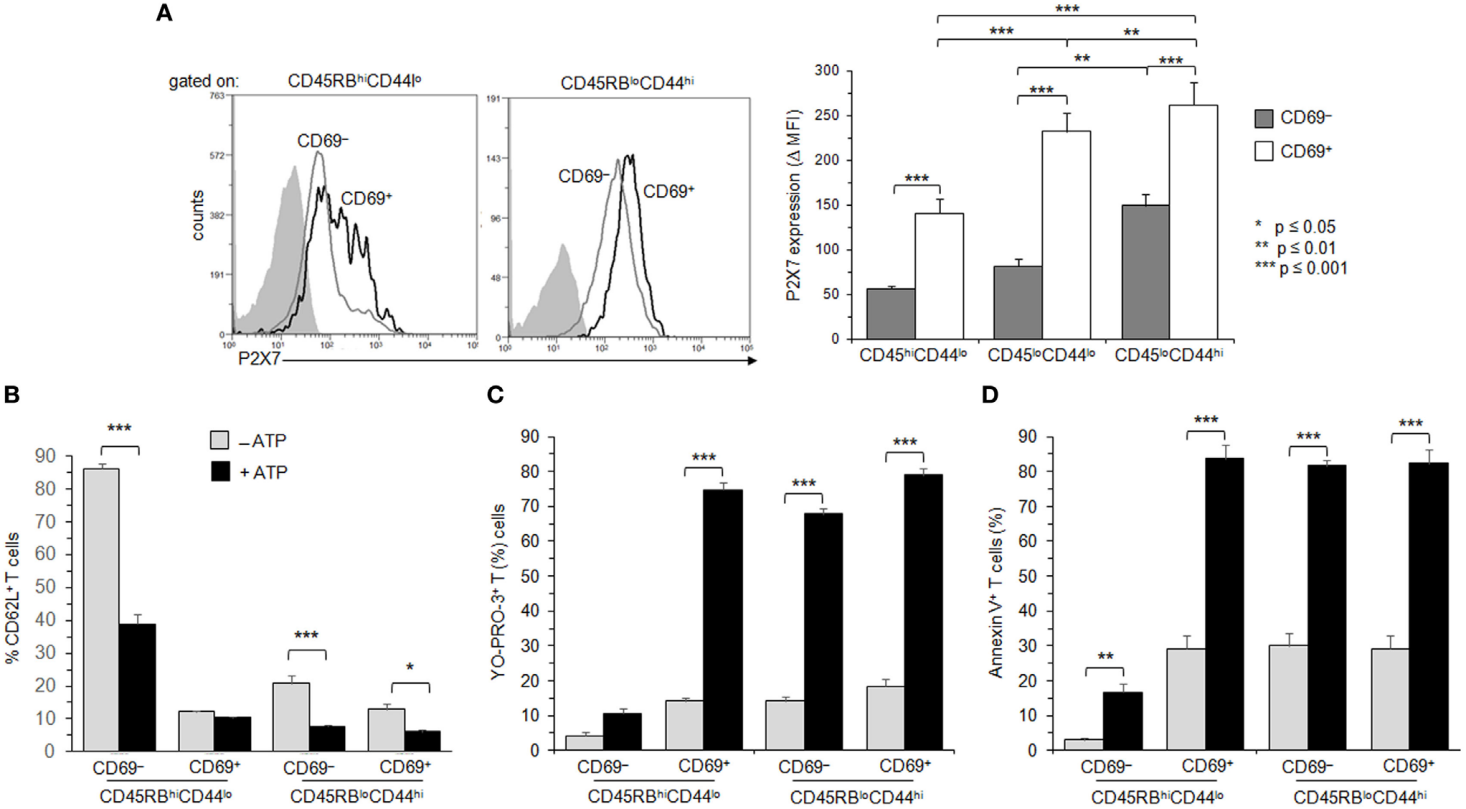
Adenosine-5′-triphosphate (ATP)-mediated cellular activities and P2X7 receptor (P2X7R) membrane expression in naive and memory T cells expressing the very early activation marker CD69. **(A)** P2X7R membrane expression was measured using rabbit polyclonal anti-P2X7R antiserum (1:100) and fluorescent-conjugated goat anti-rabbit IgG F(ab)′_2_ secondary antibodies. CD90^+^B220^−^ conventional T cells (Tconvs) from wild-type and P2X7R KO mice were separated in CD69^−^CD45RB^hi^CD44^lo^ naive, CD69^+^CD45RB^hi^CD44^lo^ recently activated naive, CD69^−^CD45RB^lo^CD44^hi^ effector/memory, or CD69^+^CD45RB^lo^CD44^hi^ recently activated memory cell subsets. In contrast with Figure 4, mice had not been injected with concanavalin A to maintain a high frequency of naive and memory resting T cells. P2X7R-staining histograms of wild-type T-cell subsets (black histograms) are overlaid on P2X7R-staining histograms of P2X7R KO T-cell subsets (gray histograms). Bar graph shows mean fluorescence intensity (MFI) ± SEM of P2X7R of 7 individual mice. Results are expressed as delta MFI (ΔMFI = MFI_wild type_ − MFI_KO_), i.e., change in MFI relative to P2X7R KO Tconvs. **(B–D)** Spleen cells from B6 mice were either left unstimulated or stimulated with 500 µM ATP for 30 min in the presence or absence of YO-PRO-3 fluorescent probe. Cells were subsequently stained with fluorescent monoclonal antibodies against phenotypic markers CD90, B220, CD69, CD45RB, CD44, and CD62L as well as Annexin V fluorescent probe. CD62L shedding, pore formation, or phosphatidylserine (PS) exposure were assessed by flow cytometry on gated naive CD45RB^hi^CD44^lo^ (either CD69^+^ or CD69^−^) and effector/memory CD45RB^lo^CD44^hi^ (either CD69^+^ or CD69^−^) CD90^+^B220^−^ T cells. The gating strategy presented in Figure S3 in Supplementary Material has been followed. Results on CD62L shedding **(B)**, pore formation **(C)**, or PS exposure **(D)** are expressed as the mean percentage ± SEM of CD62L^+^, YO-PRO-3^+^, or Annexin V^+^ cells after ATP stimulation. Asterisks denote statistically significant (*p ≤ 0.05, **p ≤ 0.01, ***p ≤ 0.001) differences between ATP-stimulated groups. Data are representative of at least six independent experiments with 7 mice per group per experiment.

**Figure 7:**
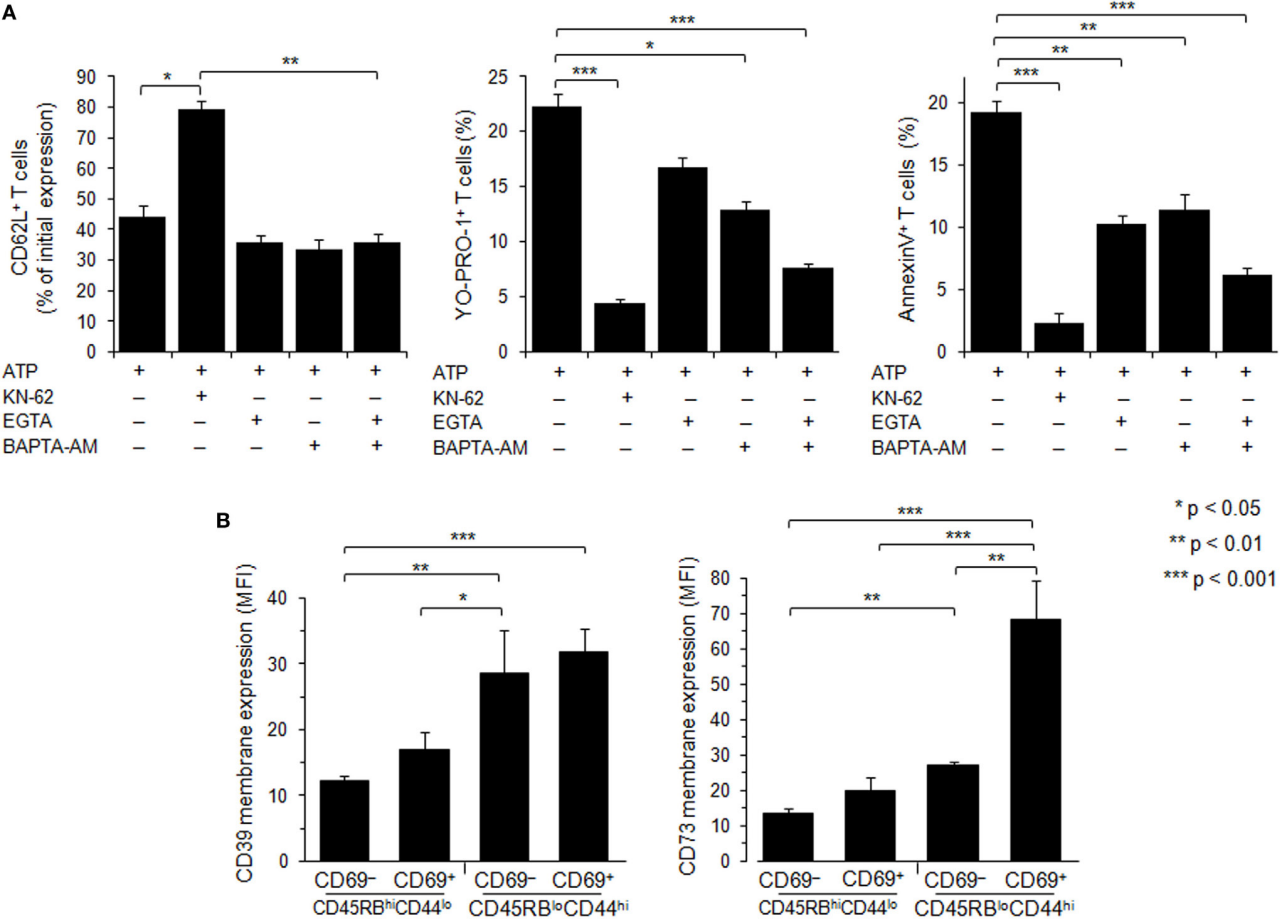
Role of calcium signaling and ectonucleotidases in P2X7 receptor (P2X7R)-dependent cellular responses in T lymphocyte subsets. **(A)** Spleen cells from B6 mice were preincubated with 5 µM P2X7R antagonist KN-62, 500 µM EGTA, 10 µM BAPTA-AM or both, for 30 min at 37°C with 5% CO_2_. Then, spleen cells were either left unstimulated or stimulated with 500 µM adenosine-5′-triphosphate (ATP) for 45 min in the presence or absence of YO-PRO-1 fluorescent probe. After washing, cells were resuspended in FACS buffer and stained with fluorescent monoclonal antibodies against phenotypic markers CD90, B220, CD69, CD44, and CD62L. After 30 min, cells were diluted in annexin-binding buffer and stained with Annexin V fluorescent probe. CD62L shedding, pore formation, or phosphatidylserine exposure were assessed within the gated CD69^−^ CD44^lo^ B220^−^CD90^+^ T-cell subpopulation by flow cytometry. Bars show the mean percentages ± SEM (*n* = 4–9 mice) of CD62L^+^ cells, YO-PRO-1^+^ cells and Annexin V^+^ cells after ATP stimulation (+) in the presence (+) or the absence (–) of KN-62, EGTA or BAPTA-AM. **(B)** CD39 and CD73 membrane expression was measured by flow cytometry on naive CD45RB^hi^CD44^lo^ and effector/memory CD45RB^lo^CD44^hi^ T cells (either CD69^−^ or CD69^+^). Mice had not been injected with concanavalin A, and the gating strategy presented in Figure S3 in Supplementary Material has been followed. Asterisks show statistically significant (*p ≤ 0.05, ** *p* ≤ 0.01, ***p ≤ 0.001) differences between ATP-stimulated groups. Data are representative of three independent experiments.

The CD69^+^CD45RB^low^ T-cell subset is heterogeneous and encompasses recently activated naive and memory T cells. Therefore, the membrane expression levels of adhesion molecule CD44, CCR7, CD45RB, CD69, and P2X7R has been used to further explore the sensitivity of naive, recently activated, effector and central memory T cells to ATP in conjunction with the levels of P2X7R membrane expression. CD4^+^ T cells (either CD69^−^ or CD69^+^) with high or low expression levels of CD45RB and CD44 naive and effector/memory markers have been identified and gated using a sequential gating strategy (Figure S3 in Supplementary Material). The differentiation of naive CD45RB^high^CD44^low^ T cells into effector/memory CD45RB^low^CD44^high^ T cells is accompanied by a significant increase in P2X7R membrane expression (Figure 6A). A further significant upregulation of P2X7R membrane expression was observed on both naive CD45RB^high^CD44^low^ and memory CD45RB^low^CD44^high^ T-cell subsets following the expression of the early activation marker CD69 (Figure 6A). Interestingly, the sensitivity to ATP of naive CD45RB^high^CD44^low^, effector memory CD45RB^low^CD44^high^ (either CCR7^−^ or CCR7^+^) and recently activated CD69^+^ (either naive or memory) T cells was not strictly correlated with the levels of P2X7R membrane expression especially for ATP-induced CD62L shedding (Figures 6B–D and data not shown). ATP-treated naive CD62L^high^CD45RB^high^CD44^low^ and effector/memory CD62L^high^CD45RB^low^CD44^high^ T cells shed efficiently CD62L (Figure 6B). The ability to shed CD62L significantly decreased after the upregulation of activation marker CD69 particularly in naive CD45RB^high^CD44^low^ T cells (Figure 6B). In contrast, naive CD45RB^high^CD44^low^ T cells had a poor ability to form pore and externalize PS after ATP treatment (Figures 6C,D). A marked upregulation of the ability to form pore and externalize PS was observed on naive CD45RB^high^CD44^low^ T cells following antigenic-activation (CD69^+^), which is retained at the effector/memory stage (Figures 6C,D). Because P2X7 displays a low affinity for ATP, we have evaluated whether the resistance to CD62L shedding observed in recently activated naive T cells upon stimulation with 0.5 mM of ATP could be overcome with higher doses of ATP. However, recently activated CD69^+^CD45RB^high^CD44^low^ T cells did not recover their ability to cleave CD62L after treatment with 1 or 2 mM ATP (data not shown). Finally, we found that calcium from the extracellular space and/or the intracellular stores was involved in pore formation and PS exposure in Tconvs, but not CD62L shedding, since they were significantly reduced in the presence of extracellular (EGTA) and/or intracellular (BAPTA-AM) calcium chelator (Figure 7A). As expected, pre-treatment of spleen cells with P2X7R antagonist KN-62 inhibited ATP-induced cellular responses (Figure 7A). Likewise, all T-cell subsets from P2X7R KO mice were resistant to 0.5–2 mM ATP stimulation, as shown by a complete lack of CD62L shedding, pore formation and PS exposure (Figure S4B in Supplementary Material). As observed in CD69^+^ T cells (Figure 4A), ADAM-17 metalloproteases were not defective in ATP-treated central memory CD62L^high^CD45RB^low^CD44^high^ T cells since PMA treatment was able to induce the cleavage of CD62L. Moreover, the shedding of CD62L induced by ATP in naive CD45RB^high^CD44^low^ T cells or by PMA in effector/memory CD45RB^low^CD44^high^ T cells could be prevented by the metalloprotease inhibitor GM6001, in a similar dose dependent-manner (Figure S4A in Supplementary Material), confirming the specificity of CD62L cleavage.

To summarize, despite high levels of P2X7R membrane expression, CD45RB^low^CD44^high^ T cells, especially upon activation (CD69^+^), are strongly resistant to ATP-induced CD62L cleavage, but not pore formation and PS exposure, demonstrating that P2X7R-mediated cellular activities vary during their activation and differentiation into effector or central memory Tconvs, independently of the levels of P2X7R membrane expression.

### CD39 and CD73 Membrane Expression on Naive, Recently Activated and Effector/Memory T Cells

Ectoenzymes CD39 and CD73 sequentially degrade extracellular ATP to adenosine. To examine whether the variation of P2X7R-mediated cellular responses of Tconvs might be due to enhanced hydrolysis of ATP following CD39 and/or CD73 overexpression, we have analyzed the levels of CD39 and CD73 membrane expression in Tconvs according to their state of activation and differentiation. Flow cytometry analyses show that the levels of CD39 and CD73 membrane expression increase with T-cell differentiation from naive CD45RB^high^ CD44^low^ to effector/memory CD45RB^low^CD44^high^ stage (Figure 7B). Interestingly, the expression of the early activation marker CD69 by memory CD45RB^low^CD44^high^ Tconvs is accompanied by a strong upregulation of CD73 (Figure 7B). However, Tconvs strongly expressing ectonucleotidases also present higher levels of ATP-induced pore formation and PS externalization. Thus, it is likely that these ectonucleotidases do not control P2X7-mediated cellular responses in Tconvs.

## Discussion

Although the ATP/P2X7R pathway is recognized as an important regulator of T cell functions (27–30), the respective sensitivities of Tregs and Tconvs to extracellular ATP are discussed and this point needs further clarification. In one report Tregs appeared to be markedly more sensitive to ATP than Tconvs, whereas in another, Tregs and Tconvs displayed similar high sensitivity to ATP provided that both T-cell populations expressed low levels of membrane phosphatase CD45RB (35, 36). However, given the large cellular and functional heterogeneity of the CD45RB^low^ Tconv population, no definitive conclusion can be drawn from these previous studies regarding the sensitivity of Tconvs to ATP. Thus, we have previously reported that effector CD45RB^low^ T cells become totally resistant to ATP at the preapoptotic stage (38). The sensitivity of T cells to ATP has also been correlated with P2X7R mRNA or protein expression levels (29, 33, 47). However, these studies were conducted in whole T-cell populations and not in T-cell subsets, which could express different levels of P2X7R depending on the stage of activation. Several reports reviewed in Ref. (48) do not show a strong correlation between mRNA expression levels and protein abundance, weakening previous conclusions on the relationship between P2X7R mRNA levels and ATP-induced cellular functions. Previous studies on the quantification of P2X7R protein expression have used Western blotting in whole-cell lysates from splenic T cells (29), which does not allow to discriminate between cell surface and intracellular P2X7R. Indeed, we have reported that P2X7R can accumulate in the cytosol of a T-cell subset without cell surface detection of P2X7R (38). Therefore, our present study aims to gain a clearer picture on the sensitivity of the major subsets of T cells to extracellular ATP in relation with the levels of P2X7R membrane expression on each subset. Through four different cellular activities (CD62L shedding, pore formation, PS externalization, cell death) triggered following the stimulation of P2X7R, we have (1) compared the sensitivity of Tconvs and Foxp3^+^ Tregs to ATP; (2) evaluated the ATP sensitivity of Tconvs at different stages of activation and differentiation; (3) quantified P2X7R membrane expression on Tconvs at different stage of activation and differentiation. In agreement with previous data (36, 37), we found that activated CD45RB^low^ Tconvs displayed significant higher sensitivity to ATP-induced PS externalization and cell death than naive CD45RB^high^ Tconvs. Moreover, we found that activated CD45RB^low^ Tconvs also displayed significant higher sensitivity to ATP-induced CD62L shedding and pore formation than naive CD45RB^high^ Tconvs (Table 1). In contrast with previous report (36), we do not find that CD45RB^low^Foxp3^+^ Tregs display higher sensitivity to ATP-induced cell death and PS externalization than activated CD45RB^low^Foxp3^−^ Tconvs (Table 1), but rather show significant higher sensitivity to ATP-induced CD62L shedding and pore formation. We suggest that the anti-CD45RB mAb used to control CD45RB expression levels in Tregs and Tconvs (36) could account for the discrepancy between this report and our present data. Indeed, we show that this mAb recognizes an epitope shared by both CD45RB and B220 molecules, suggesting that Taylor et al. (36) assessed P2X7R activity in a pool of B220^+^ and CD45RB^high^ Tconvs. Moreover, we had reported that B220-expressing effector Tconvs were totally refractory to ATP stimulation (38). Thus, in our present experiments, P2X7R-induced cellular activities were analyzed in B220-negative gated T cells. The difference in ligands used to activate P2X7R might also explain the discrepancy observed between previous studies (36) and our present data. Indeed, it has been reported that BzATP triggers pore formation in lymphocytes with an EC_50_ value of about 15 µM compared to 85 µM for ATP. Thus, BzATP stimulates P2X7-induced pore formation up to 30% more than ATP (49). In all our experiments, splenocytes have been activated with the physiological agonist ATP instead of BzATP, to avoid an overstimulation of P2X7R-mediated cellular responses that could have masked potential differences between T-cell subsets.

**Table 1:** Summary of P2X7R membrane expression and ATP-mediated cellular activities in Tconvs and Tregs.

	Tregs^a^	Tconvs^a^			
	CD45RB^lo^Foxp3^**+**^	NaiveCD45RB^hi^CD44^lo^	Activated naiveCD69^**+**^CD45RB^hi^CD44^lo^	Effector/MemoryCD69^**−**^CD45RB^lo^CD44^hi^	Activated memoryCD69^**+**^CD45RB^lo^CD44^hi^
CD62L shedding	++++	+++	−/+	+++	+
Pore formation	++++	−/+	+++	+++	+++
PS externalization	++++	++	+++	+++	+++
Cell death^b^	++++	−/+		+++	
P2X7R expression	++++	+	++	+++	++++

*^a^Treated with 0.5 mM ATP*.

*+: low level; ++: moderate level; +++: high level; ++++: very high level*.

*^b^Cell death was determined in Figure 1 (panel D) on whole CD45RB^low^ and CD45RB^high^ Tconvs in the absence of CD69 and CD44 phenotyping*.

Interestingly, we show that P2X7R-mediated cellular activities in Tconvs are not triggered in an all-or-none manner and their expressions depend on the stage of activation and differentiation. Thus, among the activated T-cell subsets, we found that recently activated CD69^+^ T cells showed a significant reduction in their ability to shed CD62L in the presence of ATP despite significant higher levels of P2X7R expression than CD69^−^ T cells. In contrast, CD69^+^ T cells display significantly higher efficiency to form pore and externalize PS than CD69-negative T cells. CD69 expression marks the activation of both naive (CD44^low^) and memory (CD44^high^) T cells. Therefore, using a more detailed phenotypic characterization of Tconvs, we have compared the levels of P2X7R membrane expression and sensitivity to ATP of naive (CCR7^+^CD45RB^high^CD44^low^) and effector/memory (CD45RB^low^CD44^high^ either CCR7^+^ or CCR7^−^), a recently activated (CD69^+^) (naive or memory) Tconvs. Naive CD62L^high^CD45RB^high^CD44^low^ Tconvs had a notably higher ability to shed CD62L in the presence of ATP than lymphoid-homing central memory CD62L^high^CCR7^+^CD44^high^ T cells, thus suggesting that P2X7R does not play a central role in the shedding of homing-receptor CD62L at least during secondary immune responses. Antigenic stimulation of naive CD45RB^high^CD44^low^ Tconvs, as evidenced by the upregulation of CD69, leads to both decreased ability to shed CD62L and increased ability to form pore (Table 1). CD62L regulates the homing of naive and central memory T cells to secondary lymphoid organs whereas sphingosine 1-phosphate receptor 1 (S1PR1) controls T cell egress from these organs. CD62L is shed from the plasma membrane of T cells following activation. The S1RP1 ligand, S1P, is expressed at low concentration in lymphoid organs and at high concentration in circulatory fluids. The S1P gradient promotes S1PR1-dependent migration of T cells from secondary lymphoid organs into the blood and lymphatic circulation. CD69 expressed on recently activated T cells causes internalization and degradation of S1PR1, delaying T cell egress (50). The shedding of CD62L on activated T cells prevents their reentry into lymph nodes, and favors the acquisition of T-cell effector functions. It is a slow process that reaches its maximum 4–6 h postactivation (51). The reduced capacity of recently activated CD69^+^ naive T cells to shed CD62L following autocrine activation of P2X7R could amplify the transient retention of recently activated T cells in the lymph nodes caused by the CD69-mediated inhibition of S1RP1, favoring a full differentiation of activated T cells into effector cells. Moreover, the higher capacity of recently activated CD69^+^ naive T cells to form pore compared to CD69^−^ naive T cells could participate to their full activation by increasing calcium entry through P2X7R acting as a costimulatory factor involved in the strength of T cell activation. Although it has been suggested that extracellular calcium is not a critical factor in pore formation (52), we found that calcium from the extracellular space and/or the intracellular stores was involved in pore formation and PS exposure in Tconvs, but not CD62L shedding. This finding agrees with our previous report showing that P2X7R-induced amyloid precursor protein shedding is independent of extracellular calcium (26). Because pore formation induces robust extracellular calcium influx (53), our data emphasize the role of P2X7R in the regulation of early signaling events involved in T-cell activation, as previously reported (27, 28, 30).

The expression of P2X7R splice variants (12, 13, 54) could account for the dissociation of ATP-induced cell activities observed during activation and differentiation of Tconvs. However, the analysis of the P2X7a and P2X7k splice variants by real time RT-PCR have shown that the Tconvs express the P2X7k variant whatever their stage of activation/differentiation (unpublished data). Membrane-bound ATPases play an important role in the regulation of cell sensitivity to ATP by regulating the extracellular concentration of ATP. Thus, ectoenzymes CD39 and CD73 sequentially hydrolyze ATP into ADP, AMP, and adenosine. However, the dissociation in ATP-induced cell activities that we observed in Tconvs is probably not linked to the levels of ATPase membrane expression. Indeed B220^+^ Tconvs, which express low levels of CD39, are totally resistant to ATP stimulation (23). Moreover, we show herein that the levels of CD39 and CD73 membrane expression increase in parallel with those of P2X7R and maximum levels of ectonucleotidases upregulation are reached on Tconvs displaying the higher levels of ATP-induced pore formation and PS externalization. Likewise, although Tregs express high levels of membrane ectonucleotidases CD39 and CD73 (55), they are highly sensitive to ATP-induced cellular functions (Table 1). The ability of Tregs to form high levels of P2X7 membrane pore might favor their regulatory functions by increasing the amount of ATP molecules released in the pericellular space, and their subsequent hydrolysis to the adenosine immunosuppressive molecule by CD39 and CD73 (55).

To summarize, CD45RB^low^ or CD25^+^ Foxp3^+^ Tregs show high levels of P2X7R membrane expression and sensitivity to ATP. In contrast, P2X7R-mediated cellular activities in CD4^+^ Tconvs are not dependent on the levels of P2X7R membrane expression and not triggered in an all-or-none manner, and depend on their stage of activation/differentiation. Thus, the P2X7R surface expression on CD4^+^ Tconvs is in the following order: CD45RB^hi^CD44^lo^ cells < CD45RB^lo^CD44^lo^ < CD45RB^lo^CD44^hi^ cells. In each of the three subsets, CD69^+^ recently activated T cells express very significantly more P2X7R than their CD69^−^ counterpart. However, the naive CD69^−^CD45RB^hi^CD44^lo^ T cells bearing the lowest surface P2X7R yield the highest CD62L shedding response but give a weak PS exposure response and do not have a significant pore formation. Recently activated CD69^+^ naive CD45RB^hi^CD44^lo^ Tconvs show a significant reduction in their ability to proteolytically cleave CD62L compared to inexperienced naive CD69^−^ T cells. The reverse situation is observed for ATP-induced pore formation, and to a lesser extent for PS externalization, which are significantly upregulated in recently activated naive CD69^+^ Tconvs compared to naive CD69^−^CD45RB^hi^CD44^lo^ Tconvs. Effector/memory CD69^−^CD45RB^lo^CD44^hi^ T cells show high levels of P2X7R membrane expression and sensitivity to ATP. Recently activated CD69^+^ effector/memory CD45RB^lo^CD44^hi^ T lymphocytes with the highest amounts of surface P2X7R respond strongly to ATP by PS exposure and pore formation but yield weaker CD62L shedding compared to their CD69^−^ counterpart.

## Ethics Statement

All the experiments were conducted in accordance with French (décret no. 2013-118) and EU (directive 86/609/EEC) guidelines for the care of laboratory animals and approved by our local research ethics committee (CEEA 59).

## Author Contributions

PB, HS, AM, and JL conceived and designed the experiments. HS, AM, JL, SL, and MB performed the experiments. PB, HS, AM, JL, SL, and JK analyzed and interpreted the data. PB and HS wrote the manuscript with the assistance of all authors. JK and CK-L critically reviewed the manuscript.

## Conflict of Interest Statement

The authors declare that the research was conducted in the absence of any commercial or financial relationships that could be construed as a potential conflict of interest.

## Supplementary Material

Figure S1 Anti-CD45RB antibodies do not distinguish CD45RB from CD45RABC (B220) isoforms. COS-7 fibroblasts were transfected with a pCDEF3 expression vector containing CD45RABC (B220) cDNA. 48 h later, transfected cells were stained with FITC-conjugated anti-CD45RA, PE-conjugated anti-CD45RB, APC-conjugated anti-CD45RC, and PE Cy5.5-conjugated anti-CD45RABC or isotype controls, and analyzed by flow cytometry. **(A)** R1 and R2 gates delineate B220-negative and B220^+^ COS-7 cells, respectively. **(B)** Expression of CD45RA, CD45RB or CD45RC in COS-7 cells gated on R2. Flow cytometry histograms obtained with anti-CD45RA, anti-CD45RB or anti-CD45RC monoclonal antibody (—) are overlaid on histograms obtained with isotype controls (- - -). At least 20,000 events were analyzed from each sample. Data are representative of three independent experiments.

Figure S2 Massive increase of CD69 expression on splenic T cells from concanavalin A (ConA)-treated mice. B6 mice were injected i.v. with 7 µg/g of T-cell mitogen ConA or phosphate-buffered saline. They were euthanized 18 h after injection, spleen cells were counted and stained with fluorescent monoclonal antibodies against phenotypic markers CD90, B220, CD4, and CD69 or isotype controls, and analyzed by flow cytometry. At least 20,000 events were analyzed from each sample. Asterisks indicate statistically significant differences between groups (****p* ≤ 0.001).

Figure S3 Representative dot plots and histograms showing sequential gating for analyses of CD62L expression levels on CD45RB^hi^CD44^lo^ naive and CD45RB^lo^CD44^hi^ effector/memory CD4^+^ T cells (either CD69^−^ or CD69^+^). Spleen cells from B6 mice were stained with either fluorescent monoclonal antibodies against phenotypic markers CD90, B220, CD4, CD45RB, CD44, CD62L, and CD69 or isotype controls, and analyzed by flow cytometry. Mice had not been injected with concanavalin A to maintain a high frequency of naive and memory resting T cells. FSC vs. SSC dot plots were used to select single, viable cells and exclude debris, dead cells and doublets. CD90 vs. B220 dot plot was used to select B220^−^CD90^+^ T cells in the FSC^hi^ SSC^lo^-gated cell population (Gate 1) and exclude B220^+^CD90^+^ T cells. The CD90^+^B220^−^ T cells were then further gated by the expression of CD4 (Gate 2). The relative expression of CD45RB and CD44 was analyzed on the CD4^+^ T-cell subset to identify CD45RB^hi^CD44^lo^ naive cells (Gate 3) and CD45RB^lo^CD44^hi^ effector/memory cells (Gate 4). Naive and memory/effector CD4^+^ T cells were then identified by the expression of CD69. Finally, the percentages of T cells expressing CD62L were gated on CD69^−^ (Gate 5) and CD69^+^ (Gate 6) cells. At least 20,000 events were analyzed from each sample.

Figure S4 Inhibition of CD62L shedding by metalloprotease inhibitor and absence of P2X7R signaling in P2X7KO mice. **(A)** Spleen cells from B6 mice (*n* = 6 mice/group) were preincubated with 0, 10, 25, 50, 100 µM of metalloprotease inhibitor GM6001 for 15 min at 37°C, and then either left unstimulated or stimulated with 2 mM adenosine-5′-triphosphate (ATP) for 30 min at 37°C. Cells were subsequently stained with monoclonal antibody (mAb) against CD62L and phenotypic markers. Cell surface expression of CD62L was assessed by flow cytometry in the gated CD90^+^, CD45RB^hi^CD44^lo^ naive, and CD45RB^lo^CD44^hi^ effector/memory CD4^+^ T cells. Bars represent the percentages of initial CD62L surface expression after ATP stimulation in the presence or absence of metalloprotease inhibitor. **(B)** Spleen cells from B6 and P2X7KO mice were either left unstimulated or stimulated with 2 mM ATP for 30 min in the presence or absence of YO-PRO-3 fluorescent probe. Cells were subsequently stained with fluorescent mAbs against phenotypic markers CD90, B220, CD4, CD45RB, CD44, and CD62L as well as Annexin V fluorescent probe. CD62L shedding, pore formation or phosphatidylserine exposure were assessed within the gated CD45RB^hi^CD44^lo^ T-cell subset by flow cytometry. Flow cytometry histograms display the staining of spleen cells from B6 or P2X7RKO mice with anti-CD62L antibody, YO-PRO-3, or Annexin V probe, left unstimulated (black line) or following ATP stimulation (gray line). Numbers inside each panel correspond to relevant cell percentages in unstimulated/stimulated conditions.

## Abbreviations

ConA, concanavalin A; mAb, monoclonal antibody; P2X7R, P2X7 receptor; PS, phosphatidylserine; Treg, regulatory T lymphocyte; Tconv, conventional T lymphocyte.
