# Supplementary figures and images for: Variations in Cellular Responses of Mouse T Cells to Adenosine-5′-Triphosphate Stimulation Do Not Depend on P2X7 Receptor Expression Levels but on Their Activation and Differentiation Stage
Source: Front Immunol. 2018 Feb 27;9:360. doi: 10.3389/fimmu.2018.00360 (PMC5835135; doi:10.3389/fimmu.2018.00360)

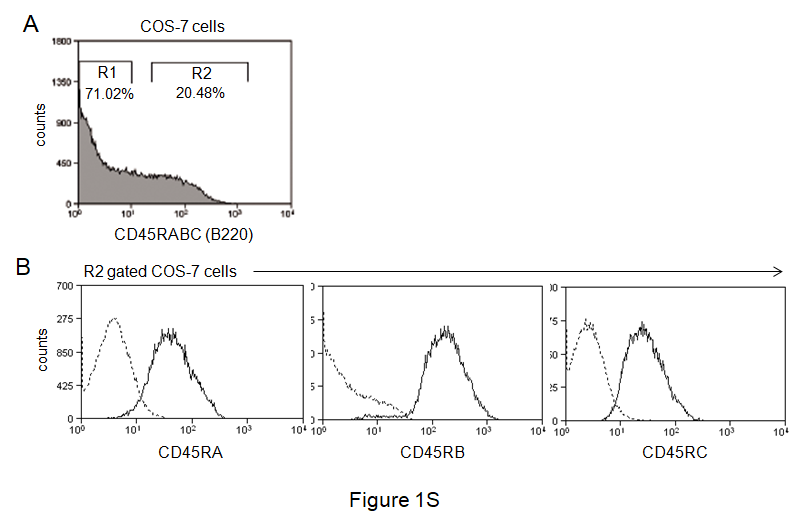

Supplement: Figure S1 — Anti-CD45RB antibodies do not distinguish CD45RB from CD45RABC (B220) isoforms. COS-7 fibroblasts were transfected with a pCDEF3 expression vector containing CD45RABC (B220) cDNA. 48 h later, transfected cells were stained with FITC-conjugated anti-CD45RA, PE-conjugated anti-CD45RB, APC-conjugated anti-CD45RC, and PE Cy5.5-conjugated anti-CD45RABC or isotype controls, and analyzed by flow cytometry. (A) R1 and R2 gates delineate B220-negative and B220+ COS-7 cells, respectively. (B) Expression of CD45RA, CD45RB or CD45RC in COS-7 cells gated on R2. Flow cytometry histograms obtained with anti-CD45RA, anti-CD45RB or anti-CD45RC monoclonal antibody (—) are overlaid on histograms obtained with isotype controls (- - -). At least 20,000 events were analyzed from each sample. Data are representative of three independent experiments. [file Image_1.tif]

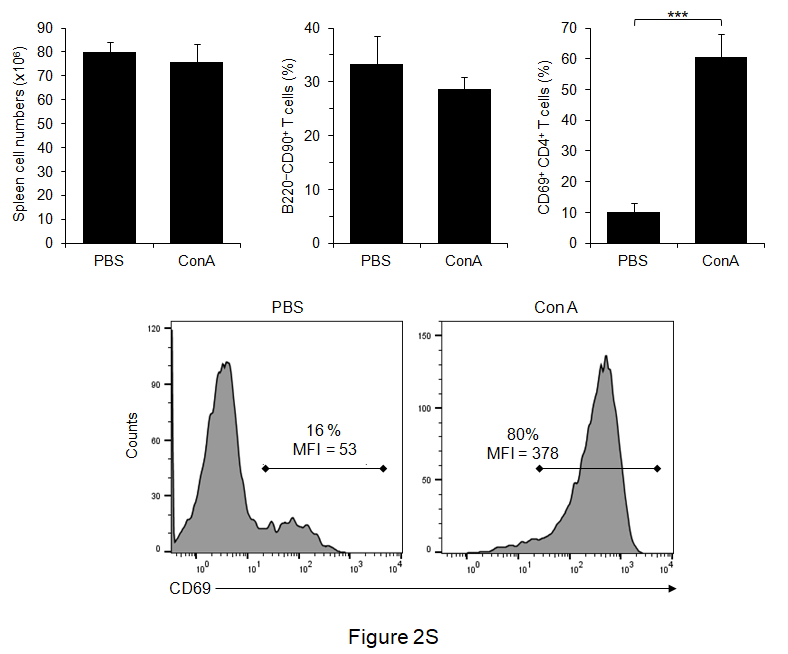

Supplement: Figure S2 — Massive increase of CD69 expression on splenic T cells from concanavalin A (ConA)-treated mice. B6 mice were injected i.v. with 7 µg/g of T-cell mitogen ConA or phosphate-buffered saline. They were euthanized 18 h after injection, spleen cells were counted and stained with fluorescent monoclonal antibodies against phenotypic markers CD90, B220, CD4, and CD69 or isotype controls, and analyzed by flow cytometry. At least 20,000 events were analyzed from each sample. Asterisks indicate statistically significant differences between groups (***p ≤ 0.001). [file Image_2.tif]

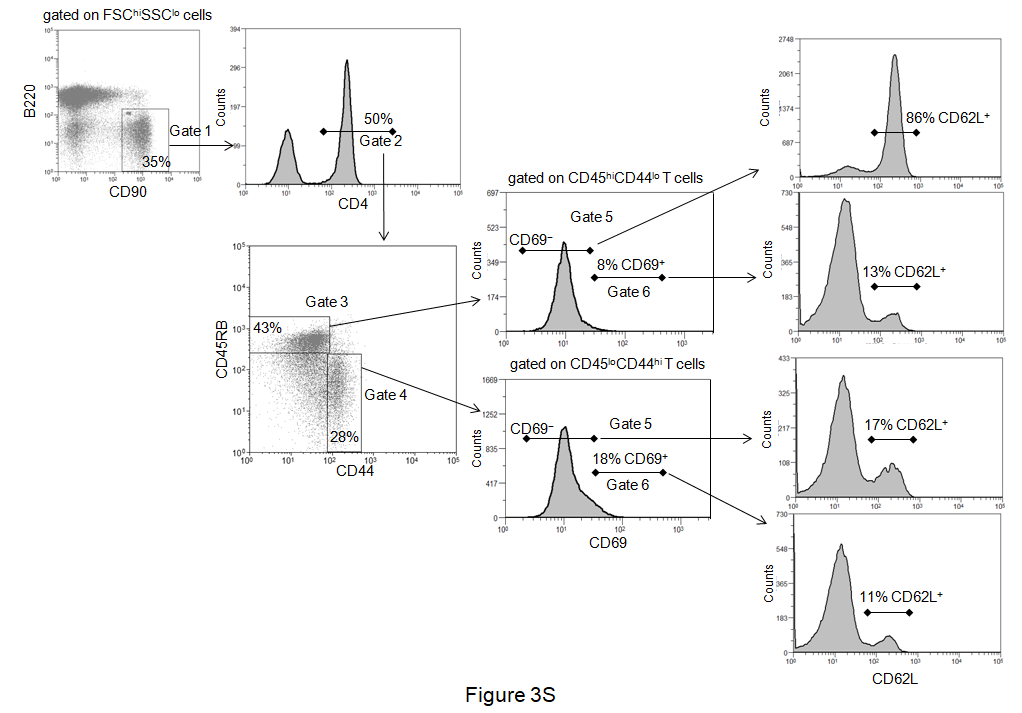

Supplement: Figure S3 — Representative dot plots and histograms showing sequential gating for analyses of CD62L expression levels on CD45RBhiCD44lo naive and CD45RBloCD44hi effector/memory CD4+ T cells (either CD69− or CD69+). Spleen cells from B6 mice were stained with either fluorescent monoclonal antibodies against phenotypic markers CD90, B220, CD4, CD45RB, CD44, CD62L, and CD69 or isotype controls, and analyzed by flow cytometry. Mice had not been injected with concanavalin A to maintain a high frequency of naive and memory resting T cells. FSC vs. SSC dot plots were used to select single, viable cells and exclude debris, dead cells and doublets. CD90 vs. B220 dot plot was used to select B220−CD90+ T cells in the FSChi SSClo-gated cell population (Gate 1) and exclude B220+CD90+ T cells. The CD90+B220− T cells were then further gated by the expression of CD4 (Gate 2). The relative expression of CD45RB and CD44 was analyzed on the CD4+ T-cell subset to identify CD45RBhiCD44lo naive cells (Gate 3) and CD45RBloCD44hi effector/memory cells (Gate 4). Naive and memory/effector CD4+ T cells were then identified by the expression of CD69. Finally, the percentages of T cells expressing CD62L were gated on CD69− (Gate 5) and CD69+ (Gate 6) cells. At least 20,000 events were analyzed from each sample. [file Image_3.tif]

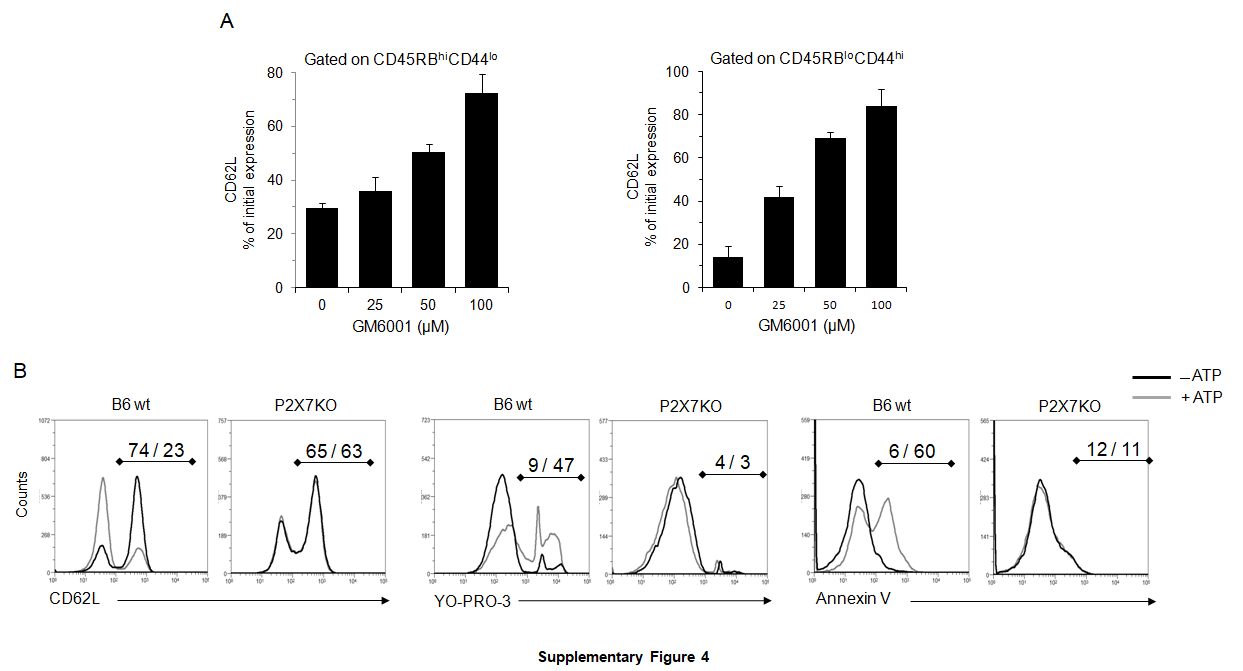

Supplement: Figure S4 — Inhibition of CD62L shedding by metalloprotease inhibitor and absence of P2X7R signaling in P2X7KO mice. (A) Spleen cells from B6 mice (n = 6 mice/group) were preincubated with 0, 10, 25, 50, 100 µM of metalloprotease inhibitor GM6001 for 15 min at 37°C, and then either left unstimulated or stimulated with 2 mM adenosine-5′-triphosphate (ATP) for 30 min at 37°C. Cells were subsequently stained with monoclonal antibody (mAb) against CD62L and phenotypic markers. Cell surface expression of CD62L was assessed by flow cytometry in the gated CD90+, CD45RBhiCD44lo naive, and CD45RBloCD44hi effector/memory CD4+ T cells. Bars represent the percentages of initial CD62L surface expression after ATP stimulation in the presence or absence of metalloprotease inhibitor. (B) Spleen cells from B6 and P2X7KO mice were either left unstimulated or stimulated with 2 mM ATP for 30 min in the presence or absence of YO-PRO-3 fluorescent probe. Cells were subsequently stained with fluorescent mAbs against phenotypic markers CD90, B220, CD4, CD45RB, CD44, and CD62L as well as Annexin V fluorescent probe. CD62L shedding, pore formation or phosphatidylserine exposure were assessed within the gated CD45RBhiCD44lo T-cell subset by flow cytometry. Flow cytometry histograms display the staining of spleen cells from B6 or P2X7RKO mice with anti-CD62L antibody, YO-PRO-3, or Annexin V probe, left unstimulated (black line) or following ATP stimulation (gray line). Numbers inside each panel correspond to relevant cell percentages in unstimulated/stimulated conditions. [file Image_4.tif]
